# The Prediction of Necroptosis-Related lncRNAs in Prognosis and Anticancer Therapy of Colorectal Cancer

**DOI:** 10.1155/2022/7158684

**Published:** 2022-09-23

**Authors:** Hanyu Xiao, Qidan Pang, Yong Wang, Suhe Lai, Hong Chen

**Affiliations:** ^1^Department of Gastrointestinal Surgery, Bishan Hospital of Chongqing Medical University, Chongqing, China; ^2^Department of Nephrology, Bishan Hospital of Chongqing Medical University, Chongqing, China

## Abstract

**Background:**

Colorectal cancer is one of the most common gastrointestinal malignancies globally. Necroptosis has been proved to play a role in the occurrence and development of the tumor, which makes it a new target for molecular therapy. However, the role of necroptosis in colorectal cancer remains unknown yet. Our study aims to build a prognostic signature of necroptosis-related lncRNAs (nrlncRNAs) to predict the outcomes of patients with colorectal cancer and facilitate in anticancer therapy.

**Method:**

We obtained RNA-seq and clinical data of colorectal adenocarcinoma from the TCGA database and got prognosis-related nrlncRNAs by univariate regression analysis. Then, we carried out the LASSO regression and multivariate regression analysis to build the prognostic signature, whose predictive ability was tested by the Kaplan-Meier as well as ROC curves and verified by the internal cohort. Moreover, we divided the cohort into 2 groups based on median of risk scores: high- and low-risk groups. By analyzing the difference in the tumor microenvironment, microsatellite instability, and tumor mutation burden between the two groups, we explored the potential chemotherapy and immunotherapy drugs.

**Results:**

We screened out 9 nrlncRNAs and built a prognostic signature based on them. With its good prognostic ability, the risk scores can act as an independent prognostic factor for patients with colorectal cancer. The overall survival rate of patients in high-risk group was significantly higher than the low-risk one. Furthermore, risk scores can also give us hints about the tumor microenvironment and facilitate in predicting the response to the CTLA-4 blocker treatment and other chemotherapeutic agents with potential efficacy such as cisplatin and staurosporine.

**Conclusions:**

In conclusion, our prognostic signature of necroptosis-related lncRNAs can facilitate in predicting the prognosis and response to the anticancer therapy of colorectal cancer patients.

## 1. Introduction

Colorectal cancer is the third most common cancer in the world [1]. There are about 900,000 deaths each year, which make it the second leading cause of cancer death worldwide [2]. Due to the lack of biomarkers for early screening and prognostic prediction, many patients are not diagnosed until to an advanced stage when they cannot be surgically treated [3]. For those patients, systemic treatments are the only choice. Although the advances in diagnosis and treatment, especially the application of molecular therapeutic targeted drugs and immune checkpoint inhibitors, have relatively extended the overall survival time of patients with advanced cancer, the overall prospect for those patients is not bright. Since immunotherapy is only effective in patients with specific genotypes [4]. Therefore, it is important to identify more novel biomarkers for diagnosis and targets for anticancer therapy.

Resistance to apoptosis is one of the main hallmarks of cancer and has long been a major impediment to anticancer therapy [5, 6]. Therefore, inducing programmed death by other mechanisms is recognized as an alternative approach to overcome this obstacle [7]. Necroptosis is a caspase-independent regulatory cell death mode [8]. It could activate the mixed lineage kinase domain-like protein (MLKL) by phosphorylation signaling pathway, which is mediated by receptor-interacting protein 1/3 (RIPK1/RIPK3) [9]. Recent studies suggest that tumor cells with resistance to apoptosis may be sensitive to the necroptosis pathway [10, 11], which is expected to be a new target for anticancer therapy. As a coalescence of necrosis and apoptosis, necroptosis is regarded to play a dual role in tumors: on one hand, necroptosis can induce “necrotic” death of tumor cells, bypassing the apoptotic pathway and elicit strong adaptive immune responses via damage-associated molecular patterns (DAMPs) to stem tumor development [12]; on the other hand, necroptosis can promote tumor metastasis and progression through destruction of endothelial cells or inflammatory response [13, 14]. Additionally, necrosis-associated inflammation enhances the immunogenicity of cancer cells, which can promisingly synergize with ICBs to create new immunotherapeutic [15].

Long non-coding RNAs (lncRNAs), defined as non-protein coding RNA transcripts of over 200 nucleotides, are engaged in various cellular biological processes, including tumor progression and immune cell infiltration [16, 17]. lncRNAs play a significant regulatory part in the development of colorectal cancer. For example, some studies showed that LINC01021 [18] could affect cell cycle, proliferation, apoptosis, epithelial-mesenchymal transformation, and other processes in colorectal cancer cells, and lncRNA-p21 [19] could inhibit the invasion and metastasis of colorectal cancer cells. Moreover, lncRNAs have also been reported to be involved in tumor cell necroptosis. Tran et al. [20] found that liver cancer cells could regulate microRNA and its target genes to induce necroptosis by expressing LINC00176. However, there are few literature reports on the role of lncRNAs in the necroptosis pathway of colorectal cancer; thus, further studies are needed.

Given that, it is necessary to shed light on interactions between necroptosis-related lncRNAs and the clinicopathological characteristics, tumor microenvironment, anticancer therapy, and tumor mutation in colorectal cancer. In summary, our work can help fill the research gap on necroptosis-related lncRNAs of colorectal cancer and provide new insights into the possible pathogenesis of colorectal cancer.

## 2. Method and Materials

### 2.1. Data Collection and Preprocessing

The RNA transcriptome data (HTSEQ-FPKM format) of colorectal adenocarcinoma were obtained from TCGA-COAD and TCGA-READ projects of the Cancer Genome Atlas (TCGA) database (https://portal.gdc.cancer.gov/) [21], including mRNA and lncRNA expression levels of tumor samples (*n* =568) and normal samples (*n* =44), as well as corresponding clinical data (XML format), such as survival information, sex, age, TNM stage, and tumor stage. We processed the data using the limma package of R language software (version 4.1.2) and Perl language (version 5.30.3) and obtained colorectal adenocarcinoma gene expression and clinical information matrix. Simple nucleotide variation data of colorectal adenocarcinoma were also downloaded from the database, and the data type was Masked Somatic Mutation, calculated by VARSCAN software (MAF format). To control the bias, patients with overall survival (OS) of less than 30 days were excluded, and 507 colorectal adenocarcinoma patients were included. We randomly divided them into the train set and test set by R caret package, with a ratio of 1 : 1. The clinical characteristics of train set, test set, and entire set are shown as Table 1.

### 2.2. Selection of Necroptosis-Related Genes and lncRNAs

We obtained 67 necroptosis-related genes (NRG) from the Gene Set Enrichment Analysis (GSEA) database (http://www.gsea-msigdb.orggseaindex.jsp) [22, 23] and previous literature [24]. Pearson's correlation analysis was performed (|Pearson R|>0.5, *P* < 0.001) to get necroptosis-related lncRNAsm (nrlncRNAs), using R limma package. We furtherly conducted the differential analysis (Log2fold change (FC)>1, false discovery rate (FDR)<0.05, and *P* < 0.05) to identify the nrIncRNAs that were significantly differentially expressed between tumor and normal samples.

### 2.3. Establishment and Validation of the Prognostic Signature

First, the necroptosis-related lncRNAs that are closely related to prognosis were screened out by univariate Cox regression analysis (*P* < 0.05). Then, to avoid overfitting and ensure the minimum amount of lncRNAs as well as complete information, the least absolute shrinkage and selection operator (LASSO) regression was performed with 10-fold cross-validation and 1000 random circles. Finally, we conducted multivariate Cox regression to identify the nrlncRNAs that could serve as independent prognostic factors for modeling. The following formula was devised based on the risk model to calculate the risk score of each patient:

(1) $$\begin{array}{c} \text{Risk score}=\overset{n}{\underset{\text{i}}{\sum }}\left(\text{Expi}*\text{Coefi}\right), \end{array}$$

where the *n* was the number of lncRNAs, Expi was the expression level of lncRNAs, and Coefi was the correlation coefficient between lncRNAs and survival data. Based on the median risk score of the train set, subgroups were established including low-risk and high-risk groups. The network between nrlncRNAs and NRGs was drawn using Cytoscape software (V3.8.0) and the survival curve and receiver operator characteristic (ROC) curve were established through R survival, survminer, and timeROC package to evaluate the predictive performance of the signature.

### 2.4. Clinical Applicability of the Signature and Construction of Nomogram

We drew the survival curves to compare the differences in survival between different clinical subgroups based on previously obtained clinical information. The risk scores were verified by univariate and multivariate COX regression to decide whether it is an independent prognostic factor. ROC curves were utilized to compare the predictive efficacy of risk scores and different clinical variables. Furthermore, the nomogram was made, using R rms package to predict the 1-, 3-, and 5-year survival rate, with age, gender, TNM stage, and risk score as variables. Meanwhile, the Hosmer-Lemeshow test was carried out to test the consistency of the predicted results with the actual situation, and decision curve analysis (DCA) was performed to compare the applicability of different clinical variables and nomograms.

### 2.5. Gene Set Enrichment Analyses

We conducted the gene enrichment analysis to uncover the biological processes that might differ significantly between the high- and low-risk groups. The Gene Ontology (GO) Gene Set (c5.go.v7.5.1.symbols.gmt) was downloaded from the Gene Set Enrichment Analysis website (http://www.gsea-msigdb.org/), including cellular component (CC), molecular function (MF), biological process (BP) gene set data, and Kyoto Encyclopedia of Genes and Genomes (KEGG) (KEGG.v7.4. symbols. GMT) gene set data. We analyzed the enrichment of two risk groups in different pathways and functions using GSEA software (version 4.1.0), whose differences were statistically significant when *P* < 0.05 and FDR <0.25.

### 2.6. Tumor Microenvironment and Immune Checkpoints

In order to know more about the tumor microenvironment (TME), we calculated the stromal score, immune score, and ESTIMATE score of the samples to deduce the tumor purity via the R estimate package. We then downloaded the immune cell infiltration files from the Timer 2.0 [25] database (http://timer.cistrome.org/). The results of the immunization assessment calculated by Timer, CIBERSORT, XCELL, QUANTISEQ, MCPcounter, and EPIC software platforms were included, and a correlation analysis was conducted between the results and risk score (*P* < 0.05). Finally, we evaluated the distribution of immune cells and immune function in two groups and compared the expression differences of immune checkpoints between the high- and low-risk groups by performing single sample Gene Set Enrichment Analysis (ssGSEA) and *T*-test. The above results were presented by box plot, bubble plot, and relationship diagram using R ggplot2, ggpubr, and packages.

### 2.7. Immunotherapy and Chemotherapeutics Sensitivity Analysis

To evaluate the performance of the prognostic signature in guiding immunotherapy, we downloaded the immunophenoscore (IPS) data of colorectal adenocarcinoma from the Cancer Immune Altas (TCIA, https://tcia.at/home) [26] database to compare the responses of patients to PD-1 and CTLA-4 blockers treatment in high- and low-risk groups. Moreover, we compared the half-maximal inhibitory concentration (IC50) of different chemotherapy drugs to identify the promising therapeutic substances, of which data were obtained from Genomics of Drug Sensitivity in Cancer database (GDSC) (https://www.cancerrxgene.org/) and analyzed by oncoPredict package [27].

### 2.8. Microsatellite Instability and Tumor Mutation Burden

Similarly, we obtained the data related to microsatellite instability (MSI) in patients with colorectal adenocarcinoma from the TCIA database and used R ggplot package to draw the percentage chart of microsatellite stability (MSS), MSI-L, and MSI-H in high- and low-risk groups. A waterfall map was drawn through R maftools package to display the mutation frequency and mutation type of tumor mutation genes in the two risk groups based on the previously downloaded data. Ultimately, we compared the tumor mutation burden (TMB) and its effect on the prognosis by difference analysis and survival curve. TMB is determined by the ratio of the frequency of somatic mutations to the length of exon effective regions [28], and the somatic mutation data were derived from TCGA-COREAD mutation data.

## 3. Results

### 3.1. Identification of Necroptosis-Related lncRNAs

**Figure 1:**
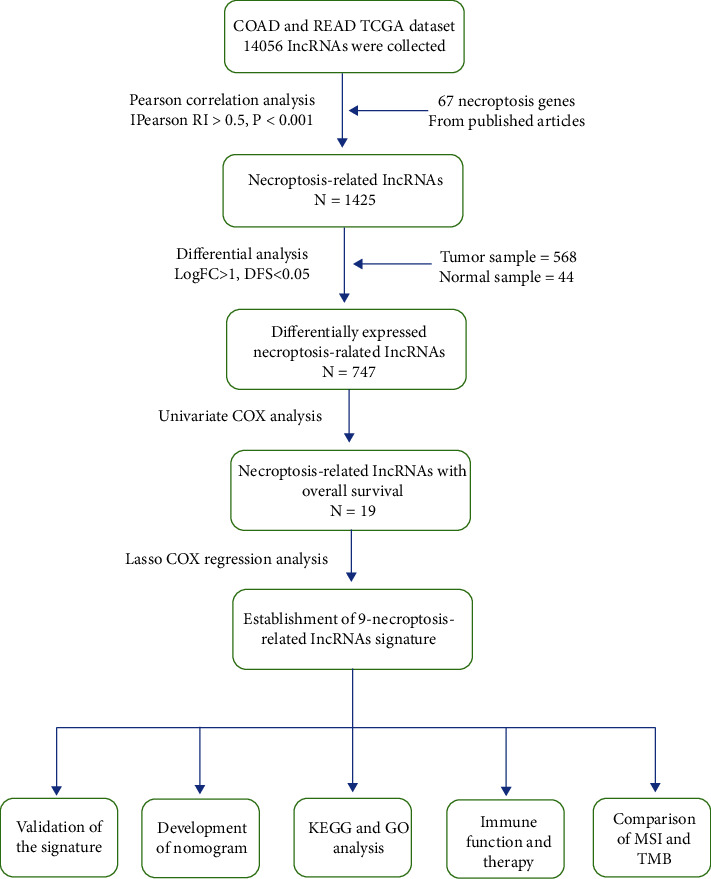
The main analysis process of this study.

**Figure 2:**
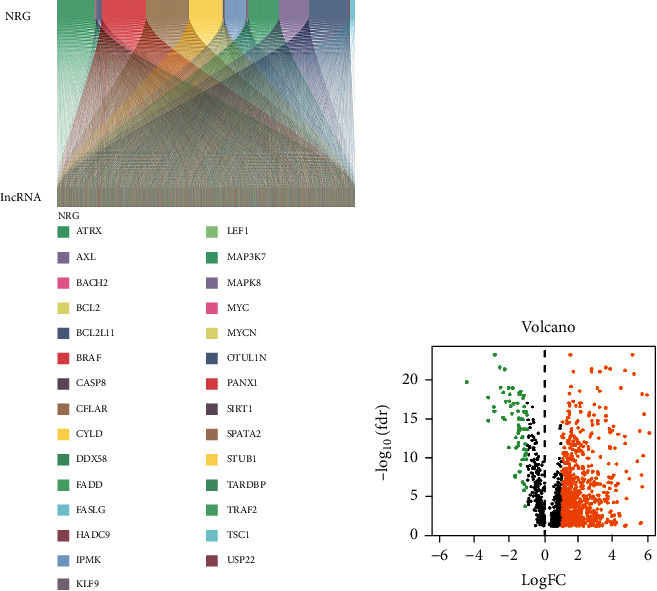
Extraction of necroptosis-related lncRNAs in CRC patients. (a) The Sankey diagram of necroptosis-related genes (=67) and related lncRNAs (=1425) (correlation coefficients >0.5 and *P* < 0.001). (b) The volcano plot of 747 differentially expressed necroptosis-related LncRNAs. (LogFC>1 and DFS<0.05).

The entire analysis process is shown in Figure 1. We obtained 568 colorectal adenocarcinoma samples and 44 normal samples from the TCGA database and sorted out the expression matrix of 56461 lncRNAs and 67 necroptotic-related genes (NRG) corresponding to each sample. 1425 necroptosis-related IncRNAs (nrlncRNAs) were identified by Pearson's correlation analysis (|Pearson R|>0.5, *P* < 0.001). The Sankey diagram (Figure 2(a)) showed the connections between 67 NRGs and nrlncRNA gene sets. Through differential analysis (LogFC>1, DFS<0.05), we found 747 significantly differentially expressed nrlncRNAs between tumor and normal samples. The volcano plot (Figure 2(b)) showed that 679 nrlncRNAs were significantly up-regulated and 68 nrlncRNAs significantly down-regulated in tumor samples.

### 3.2. Establishment and Verification of Prognostic Signature

**Figure 3:**
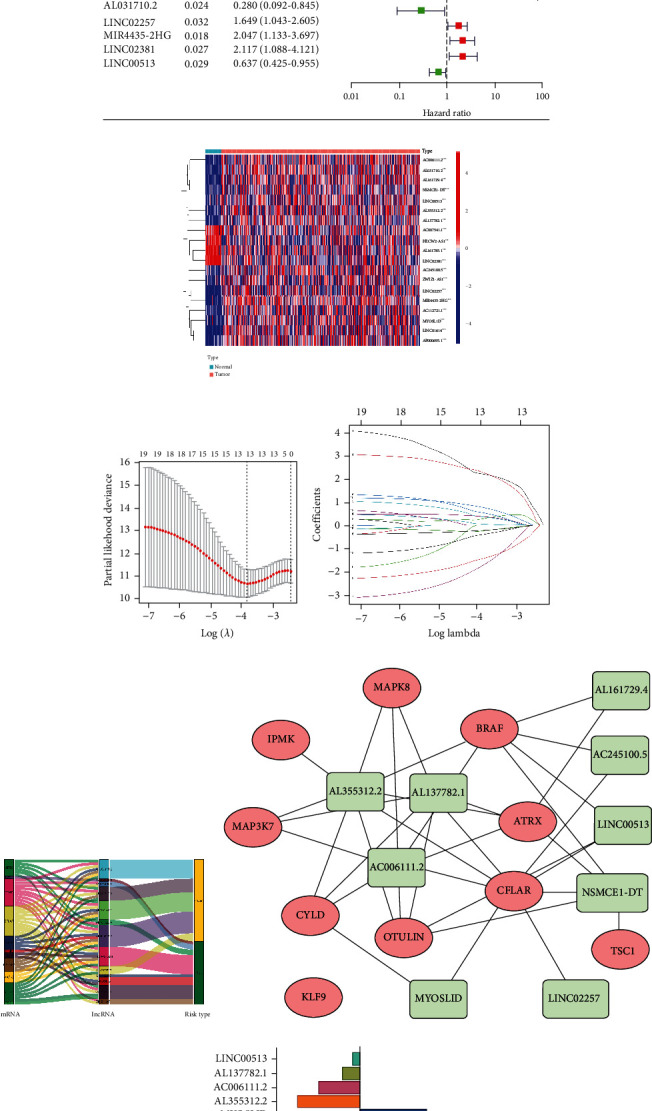
Construction of necroptosis-related lncRNAs' prognostic signature for CRC. (a) The forest map of prognostic nrlncRNAs extracted by univariate Cox regression analysis. (b) The expression profiles of 19 prognostic nrlncRNAs. (c) The partial likelihood deviance plot presented the minimum number corresponding to the covariates used for multivariate Cox analysis. (d) LASSO coefficient profiling of the 13 nrlncRNAs. (e) The Sankey diagram of the relationship between NRG, nrlncRNAs, and risk type. (f) Network showed the interaction of 9 nrlncRNAs for signature and NRG. (g) The column diagram showed the 9 nrlncRNAs for signature and corresponding coefficients.

In order to screen out nrlncRNAs that can predict the prognosis of patients, we used univariate COX regression analysis to obtain 19 nrlncRNAs that significantly affected the overall survival rate (OS) of patients (all *P* < 0.05). The forest map (Figure 3(a)) showed the hazard ratio (95% confidence interval) of these 19 nrlncRNAs, and the heat map (Figure 3(b)) reflected their expression levels in tumor or normal samples. To reduce the overfitting combination of linear regression and improve the model's accuracy, we adopted LASSO regression to reduce dimensionality and got the variables corresponding to the minimum partial likelihood deviance (Figure 3(c)), namely, the identified 13 nrlncRNAs. Through multivariate COX regression, 9 nrlncRNAs (Figure 3(g)) that could be used as independent prognostic factors were finally obtained for modeling. The Sankey diagram (Figure 3(e)) and network diagram (Figure 3(f)) showed the corresponding connections between these nrlncRNAs and necroptosis mRNAs. The risk scores based on the expression level of nrlncRNAs was as follows: Risk Score = MYOSLID expi× (3.2358) + AC006111.2 expi× (-1.9913) + AC245100.5 expi × (0.5341) + AL161729.4 expi × (1.1819) + AL355312.2 expi × (-3.0215) + AL137782.1 expi × (-0.8773) + NSMCE1-DT expi × (2.9534) + LINC02257 expi × (0.8564) + LINC00513 expi × (-0.3655).

**Figure 4:**
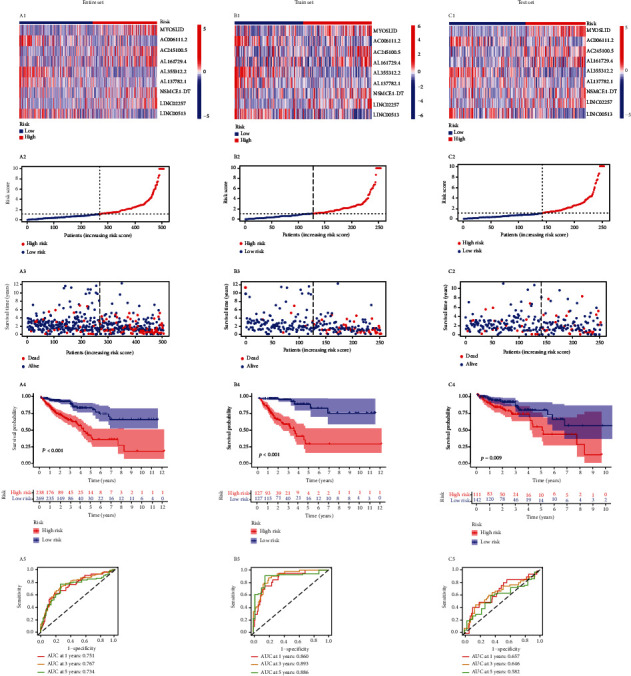
Validation of necroptosis-related lncRNAs' prognostic signature for CRC. (a1–a5) Expression of 9 nrlncRNAs for signature in high- and low-risk groups, the distribution of the risk score, survival status, the Kaplan–Meier survival curve, and the time-dependent ROC curve analyses of the prognostic signature in the entire set. (b1–b5) The demonstration mentioned above in the train set. (c1–c5) The demonstration mentioned above in the test set.

The median risk score of patients in the train set acted as the node to divide the cohort into high- and low-risk groups, while the test set was used for in-cohort verification.  Figure 4 shows the nrlncRNAs expression level, risk score, and survival time distribution of high- and low-risk groups in the entire set, train set, and test set. Survival curve indicated that overall survival rate in the high-risk group was significantly lower than low-risk group (entire set: *P* < 0.001, train set: *P* < 0.001, test set: *P* = 0.009). The ROC curve showed that the one-year prognosis area under the curve (AUC) was 0.751, 0.860, and 0.657, three years 0.767, 0.893, and 0.646, and five years 0.751, 0.860, and 0.657 in the three cohort sets, respectively. In addition, the cohort was divided into 9 pairs of high- and low-expression groups based on each of the selected nrlncRNAs, respectively, and 7 nrlncRNAs showed significant differences in overall survival rate (Supplementary 1).

### 3.3. Clinical Applicability of Prognostic Signature

**Figure 5:**
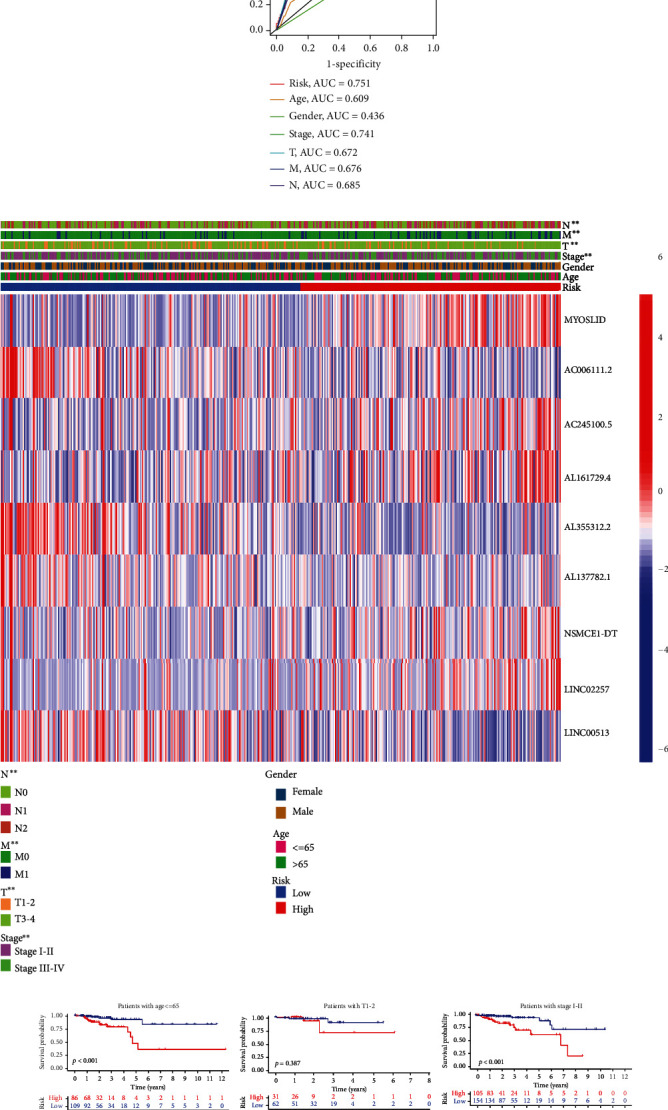
Clinical applicability of prognostic signature. (a, b) Univariate and multivariate Cox regression analysis of the correlation between the risk scores and clinicopathological features. (c) ROC curve analyses of the risk scores and clinicopathological features. (d) The heat map and clinicopathological factors of high- and low-risk subgroups. ^∗^*P* < 0.05, ^∗∗^*P* < 0.01, ^∗∗∗^*P* < 0.001. (e) The Kaplan–Meier survival analyses of prognostic signature in different clinical subgroups based on age, gender, stage, grade, and TMN stage with the log-rank test.

It was found that risk scores could serve as an independent risk factor for prognosis among clinical factors including age, gender, tumor stage, and TNM stage (Figures 5(a) and 5(b)), by univariate COX regression analysis and multivariate COX regression analysis, with respective hazard ratio 1.055 (95% CI: 1.039-1.072) and 1.051 (95% CI: 1.034-1.069). ROC curve (Figure 5(c)) showed that the area under the signature curve was 0.751, greater than the 0.741 of the clinical staging, which was widely used as a prognostic predictor in clinical practice. According to the heat map (Figure 5(d)), the expression levels of nrlncRNAs for the signature varied greatly in different tumor stages or TNM stages. Furthermore, we compared the Kaplan-Meier curves of the high- and low-risk groups under different clinical conditions (Figure 5(e)) and found that the overall survival rate of the high-risk group was far worse than that of the low-risk group, except in the case of early T stage (T1-T2) and distant metastasis (M1), possibly due to the insufficient number of patients. All this indicated that the prognostic signature applied to patients with different clinical conditions.

### 3.4. Construction and Verification of Nomogram

**Figure 6:**
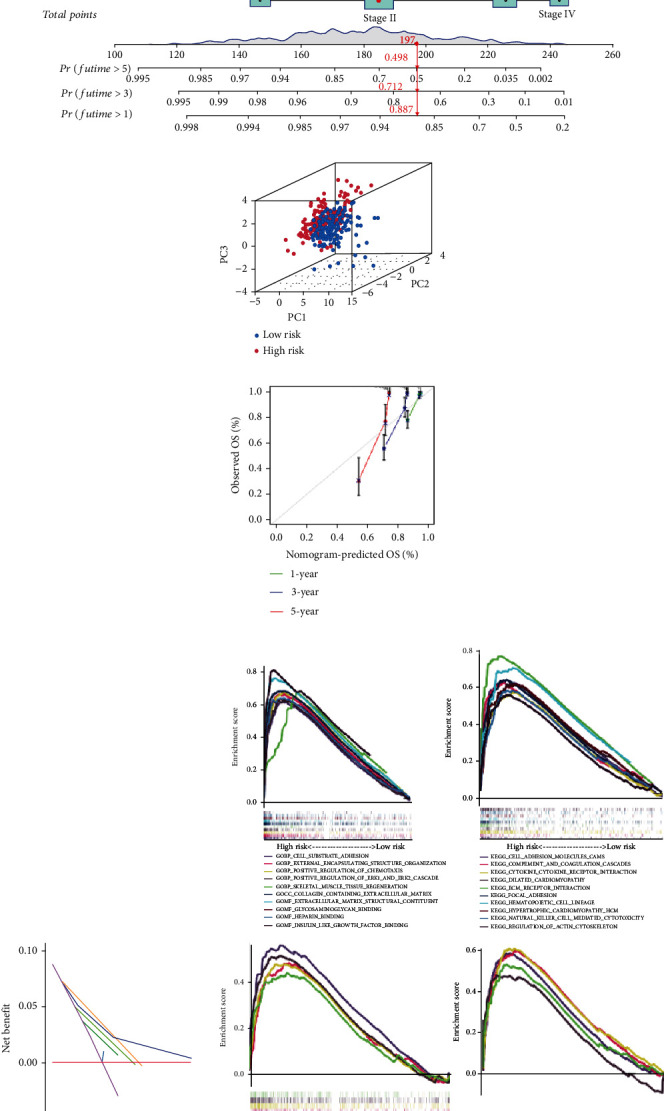
Establishment and validation of the nomogram. (a) The nomogram for predicting the 1-, 3-, and 5-year OS of CRC in the cohort based on the risk score and other clinical factors. (b) Principal component analysis (PCA) plot for nrlncRNAs. (c) Calibration plots for assessing the accuracy of the 1-, 3-, and 5-year survival rates. (d) Decision curve analysis (DCA) diagram of nomogram and other clinical factors. (e) GSEA of the top 10 pathways of GO and KEGG database significantly enriched in the high-risk group and of the tumor-associated and immune-associated pathways significantly enriched in the high-risk group.

Using variables such as age, sex, clinical stage, TNM stage, and risk score, we constructed a nomogram that predicted 1-, 3-, and 5-year survival. In Figure 6(a), a 67-year-old female CRC patient with stage T3N0M0 II was grouped into the high-risk group with a total score of 197. The predicted 1-year survival rate of this patient was 88.7%, 3-year survival rate 71.2%, and 5-year survival rate 49.8%. Principal component analysis (PCA) diagram (Figure 6(b)) showed that it is easy to distinguish the patients between high- and low-risk groups. We also used 1-, 3-, and 5-year calibration curves (Figure 6(c)) to demonstrate that the nomograms were broadly consistent with the observed OS. Decision curve analysis (Figure 6(d)) showed that the nomogram was more effective than other clinical factors in predicting patient outcomes.

#### 3.4.1. Gene Set Enrichment Analysis

Compared with the low-risk group, genes in the high-risk group were significantly enriched in multiple biological functions and pathways, including extracellular matrix structure and some inflammatory pathways (Figure 6(e)), which justified the necessity of further research in the tumor microenvironment and associated immunity. However, no significant gene enrichment was found in the low-risk group. Additionally, we found that some high-risk genes were significantly enriched in tumor pathways and immune pathways, such as MAPK, Wnt, TGF-*β*, Toll-like receptor signaling pathway, and the like.

### 3.5. Tumor Microenvironment and Immune Infiltration

**Figure 7:**
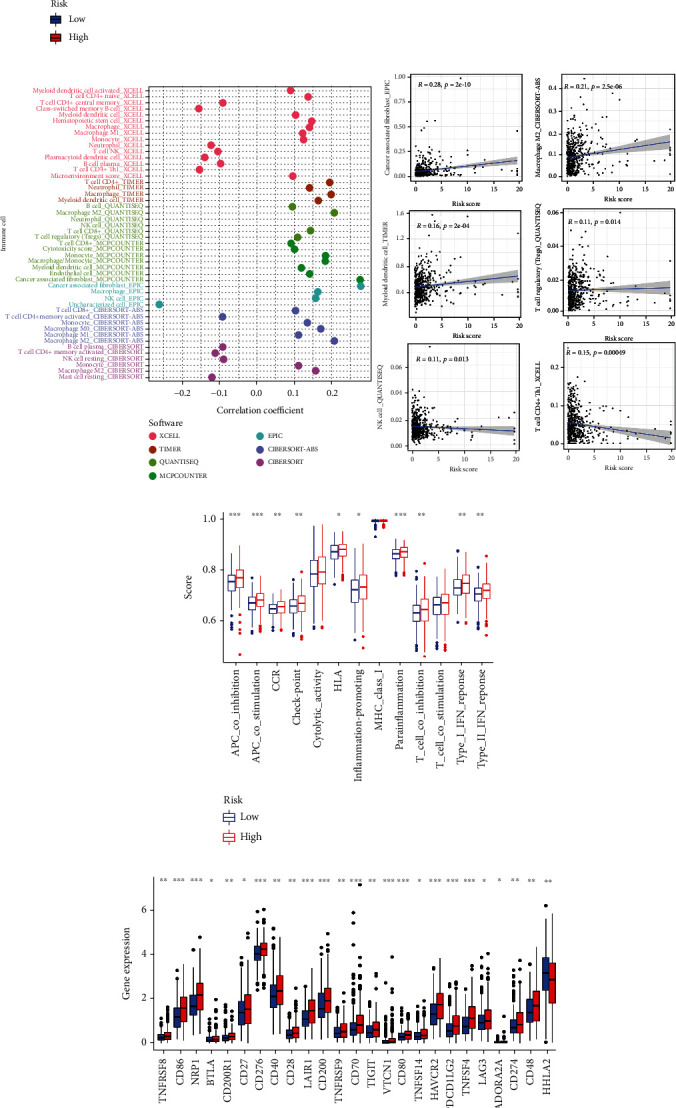
The investigation of tumor microenvironment and function. (a) The comparison of the stromal score, immune score, and ESTIMATE score between low- and high-risk groups. (b) The bubble and scatter diagrams showed correlation between risk scores and immune cells infiltration. (c) The box plot displayed the differences of enrichment scores 13 immune-related pathways in high- and low-risk groups. (d) The box plot exhibited the differences of 15 checkpoints expression in risk groups.

Using the ESTIMATE algorithm, we found that the stromal score, immune score, and estimate score in the high-risk group were significantly higher than those in the low-risk group (Figure 7(a)). In terms of immune cell infiltration (Figure 7(b)), the expression data of immune cells on different software platforms showed that the number of most immune cells infiltrated was positively correlated with risk scores, including cancer-associated fibroblast (EPIC), Macrophage M2 (CIBERSORT_ABS), T Cell Regulatory (Tregs) (QUANTISEQ), Myeloid Dendritic Cell (TIMER), and the like, while the number of some immune cells infiltrated was negatively correlated with the risk scores, such as NK cell (QUANTISEQ) and T cell CD4+ Th1 (XCELL). Similarly, ssGSEA scores (Figure 7(c)) of multiple immune functions were significantly higher in the high-risk group than in the low-risk group.

### 3.6. Immune Checkpoints and Anticancer Therapy

**Figure 8:**
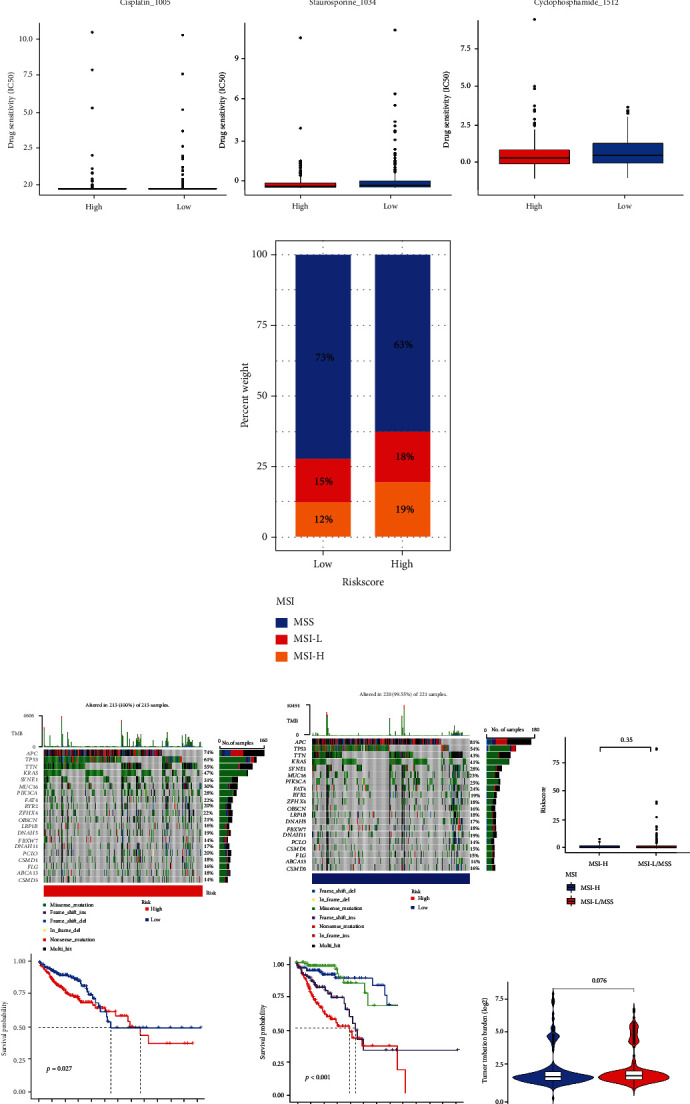
Response to immunotherapy and evaluation of microsatellite instability and tumor mutation burden among high- and low-risk groups. (a) The association between IPS and risk groups for CRC patients. (b) The chemotherapy prediction of risk groups through IC50 of drugs. (c) Distribution of MSS, MSI-L, and MSI-H patients between high- and low-risk groups, and comparison of risk scores in MSI-H and MSS/MSI-L groups. (d) Mutation profiles of high- and low-risk groups, survival analysis associated with TMB and risk groups, and differences of TMB between high- and low-risk groups.

The expression levels of 24 immune checkpoints were significantly higher in the high-risk group than the low-risk group (Figure 7(d)), including CD274 (PD-1) and other novel immune checkpoints such as NRP1, BTLA, TIGIT, and VTCN1. It indicated that the high-risk group might have greater immunotherapy promises. As shown in Figure 8(b), the half-maximal inhibitory concentration (IC50) of therapeutic substances in the high-risk group, including cisplatin, staurosporine, and cyclophosphamide, was notably lower than that of the low-risk group, which showed that patients in the high-risk group were more sensitive to these compounds. However, there were no significant differences in drug sensitivity to 5-fluorouracil, oxaliplatin, and irinotecan between the two groups. Furthermore, by comparing the immunophenoscore (IPS) of two groups when treated with PD-1 blocker and CTLA-4, we found that the low-risk group had a better response to the latter, while no significant difference was observed in response to PD-1 blocker or the combination of the two between the two groups.

### 3.7. Microsatellite Instability and Tumor Mutation

Compared with the low-risk group, the high-risk group had larger proportions of microsatellite instability-high (MSI-H), microsatellite instability-low (MSI-L) (19% vs.12%, 18% vs.15%), and lower microsatellite stability (MSS) (63% vs.73%) (Figure 7(c)), while there was no significant difference in risk scores between MSS/MSI-L and MSI-H patients. In the two risk groups, APC, TP53, TTN, KRAS, and SYNE1 were the top five mutation genes, of which the proportion of APC was higher in the low-risk group, and the proportion of TP53, TTN, KRAS, and SYNE1 in the high-risk group was higher (Figure 7(d)). The high-risk group's tumor mutation burden (TMB) was higher than that of the low-risk group, although there was no significant statistical difference. The TMB remarkably impacted the overall survival rate. Survival was worse in the TMB-H group than TMB-L group at 1 to 5 years, but at 5 to 7 years, it was the other way around. However, the effect appeared weaker than risk scores, for patients in the high-risk group had much worse outcomes than those in the low-risk group, even at a low tumor low mutation burden.

## 4. Discussion

Colorectal cancer is a highly malignant digestive system tumor with high morbidity and mortality. Although a systemic treatment protocol has been formed on the basis of surgery, chemotherapy, and radiotherapy, the prognosis is still not optimistic. One of the reasons is chemotherapy resistance, directly or indirectly [29]. Necroptosis is considered to be an alternative for apoptosis, which plays a role in inducing necrotic cell death when tumor cells develop drug resistance due to dysregulation of the apoptosis mechanism [30]. Moreover, more and more studies have shown that necroptosis also got involved in tumorigenesis.

Given that, Huang et al. [31] used necroptosis-related miRNAs to predict the prognosis of colon cancer patients, which have shown fair performance. However, we only have a limited understanding of the interactions between lncRNAs and necroptosis in colorectal cancer, which requires further study. This study identified 9 necroptosis-related lncRNAs (nrlncRNAs) from the public database, established a prognostic signature, and grouped them by risk scores to explore the signature's performance in predicting immune infiltration and guiding immunotherapy. Our study suggested that prognostic signature based on necroptosis-related lncRNAs can be used for prognostic stratification of colorectal cancer patients and help to decipher the molecular mechanism of colorectal cancer.

Previous researches have proved that necroptosis-related lncRNAs are closely related to gastric cancer patient's prognosis and can be used to distinguish between cold and hot tumors and guide immunotherapy [24]. This has demonstrated nrlncRNAs' research potential in gastrointestinal tumors. Through statistical analysis and screening, we managed to obtain 9 nrlncRNAs that can be utilized to predict prognosis, including MYOSLID, AC006111.2, AC245100.5, AL161729.4, AL355312.2, AL137782.1, NSMCE1-DT, LINC02257, and LINC00513, of which the former five lncRNAs were risk factors and the latter four were protective factors. MYOSLID, with the highest hazard ratio, has the most significant correlation with survival rate (coefficient =3.236, hazard ratio =16.54 (2.92-93.64), *P* = 0.0020), which has been reported to regulate the biological processes of various tumor cells. Han et al. [32] demonstrated the critical role of the MYOSLID-miR-29c-MCL-1 axis in the tumorigenesis of gastric cancers. Xiong et al. [33] found that MYOSLI can promote the invasion and metastasis of squamous cell carcinomas of the head and neck by regulating epithelial-mesenchymal transformation. Moreover, LINC02257 [34] has also been used for predicting the outcomes of colorectal cancers, whose expression level is associated with the immune state. LINC00513 [35] is considered to play a part in systemic lupus erythematosus and acts as a positive regulator of interferon signaling pathways. Other nrlncRNAs are not reported in relevant literature, whose biological effects need to be explored. There are close interactions between these nrlncRNAs and necroptotic genes, indicating that they share some common mechanisms in necroptotic pathways. Although the expressions of these lncRNAs are all significantly positively correlated with necroptotic genes, they have different effects on prognosis, which is consistent with the contradiction of necroptosis in tumors.

The prognostic signature based on these nrlncRNAs has good predictive performance. Clinically, the most widely used prognostic indicators of colorectal cancer are TNM stage and clinical stage, while our signature is superior to these indicators and applicable to different ages, genders, and stages, whose effectiveness is furtherly verified by the internal cohort. Besides, we also drew a nomogram that included risk scores and clinical indicators to facilitate the prediction of the 1-, 3-, and 5-year survival rate of a single patient, whose prediction results had good consistency with the actual situation.

Gene enrichment analysis revealed that genes in the high-risk group were significantly enriched in common signaling pathways of tumors and immune signaling pathways, which may be the underlying molecular mechanisms behind the worse prognosis of patients in the high-risk group. In addition, genes in the high-risk group are also involved in extracellular matrix related biological processes. Extracellular matrix is a protein network surrounding normal cells and cancer cells, acting as an important component of tumor microenvironment, which greatly influences tumor proliferation, angiogenesis, adhesion, movement, invasion, and metastasis [36, 37]. Thus, it is necessary to probe into the tumor microenvironment.

Immune cell infiltrated in tumor tissue could influence the onset and development of tumor and its response to immunotherapy [38–41]. Overall, the high-risk group is burdened with higher tumor cell purity and more immune cell infiltration than the low-risk group. Specifically, the infiltration of M2-type macrophages, Tregs, and dendritic cells is significantly positively correlated with the risk scores. Studies have shown that massive M2-type macrophages in tumor tissues signified poor prognosis [42]. On one hand, Tregs cells exert greater immunosuppressive function by upregulating the expression of cytotoxic T lymphocyte antigen-4 (CTLA-4) and programmed death receptor-1 (PD-1) in colorectal cancer [43]. On the other hand, the migration of T cells to the tumor is inhibited by Treg cells by regulating chemokines [44] and secreting cytokines that block antitumor immunity [45]. The degree of dendritic cell infiltration is positively correlated with the metastasis and stage of colorectal cancer [46, 47]. In part, NK cells and Th1 cells are negatively correlated with the risk scores, which perform an antitumor function [48, 49].

Immune checkpoint inhibitors (ICIs) enhance the cytotoxicity effect of T cells on tumor cells by acting on co-inhibitory receptors such as CTLA-4 and PD-1 or their ligands such as programmed death ligand-1 (PD-L1) [50]. The application of ICIs in colorectal cancer is beneficial in a minority of patients with high immunogenicity. In our study, the low-risk group had better response to the CTLA-4 blocker treatment, even though there is a larger proportion of MSI-H patients. In the high-risk group, more immune checkpoint genes were expressed. The high exposure of these immune checkpoints and infiltration of M2 macrophages, Treg cells, and MODS cells constitute the immunosuppressive environment in the high-risk group. Research has shown that necroptosis-induced CXCL1 and Mincle signaling promote macrophage-induced adaptive immune suppression enabling pancreas oncogenesis [51].

Therefore, the five lncRNAs highly expressed in the high-risk group may be involved in the regulation of necroptosis-induced immunosuppression in colorectal cancer, which may account for the patients' relative insensitivity to ICIs in the high-risk group.

Recent studies have found that some compounds can play a role in anticancer therapies by inducing necroptosis, which may provide us a new promising therapeutic approach for bypassing the acquired or intrinsic apoptosis resistance. For example, cisplatin can induce necroptosis in esophageal and lung cancer cells [52, 53] without being affected by apoptosis resistance. Staurosporine has been reported to induce RIPK1 and MLKL-dependent necroptotic cell death in leukemia cells when caspase activation is compromised [54]. In this study, the patients in the high-risk group were more sensitive to cisplatin and staurosporine, which implies the value of both compounds in the treatment of colorectal cancer and the role of lncRNAs in the regulation of necroptosis induction. However, the guidance for the use of chemotherapy drugs including 5-fluorouracil, oxaliplatin, irinotecan, and raltitrexed is limited, which are commonly used in colorectal cancer.

Tumor mutations are not rare in colorectal cancers, and the tumor cells with a high tumor mutation burden (TMB) express more tumor antigens, which intensifies the immune-killing effect [55]. However, TMB did not differ significantly between the high- and low-risk group, suggesting similar immunogenicity due to mutation exposure between the two groups. Moreover, the tumor mutation burden also has an impact on prognosis. Studies [56] have shown that patients with high TMB have a longer overall survival time compared with colorectal cancer patients with low TMB, which is consistent with the long-term survival prospects in our cohort.

Our study had some limitations: Firstly, the prognostic signature and the nomogram lacked external queue verification [57]. We tried to download gene chips from the Gene Expression Omnibus (GEO) database for external verification, but the expression level of key lncRNAs could not be extracted due to the incomplete sequencing genes. Secondly, only limited data analysis cannot fully elucidate the specific regulatory role of 9 nrlncRNAs in necroptosis of colorectal cancer and further exploration in vivo and in vitro experiments is needed. Finally, our data mainly came from the TCGA database, and the missing data is not random, which may cause some bias.

## 5. Conclusion

In conclusion, our study established a relatively reliable prognostic signature on the basis of necroptosis-related lncRNAs, which facilitates in predicting the prognosis as well as tumor immune microenvironment, and guiding the antitumor treatment of colorectal cancer patients according to the risk stratification. Our study also showed that lncRNAs may get involved in the double nature of necroptosis in colorectal cancer, and its molecular mechanisms require further experiment verification.

## Figures and Tables

**Table 1:** The clinical information in train set, test set and entire set.

Characteristic	Type	Entire set	Train set	Test set
Age	≤65	225 (44.4%)	103 (40.6%)	122 (48.2%)
	>65	282 (55.6%)	151 (59.4%)	131 (51.8%)
Sex	Male	227 (54.6%)	135 (53.1%)	142 (56.1%)
	Female	230 (45.6%)	119 (46.9%)	111 (43.9%)
Stage	Stage I-II	279 (55.0%)	145 (57.1%)	134 (53.0%)
	Stage III-IV	213 (42.0%)	100 (39.4%)	113 (44.7%)
	Unknown	15 (3.0%)	9 (3.5%)	6 (2.3%)
T	Tis	1 (0.2%)	0 (0)	1 (0.4%)
	T1-2	105 (20.7%)	53 (20.9%)	52 (20.6%)
	T3-4	401 (80.1%)	201 (79.1%)	200 (79.0%)
N	N0	296 (58.4%)	152 (59.8%)	144 (56.9%)
	N1	124 (24.4%)	67 (26.4%)	57 (22.5%)
	N2	86 (17.0%)	35 (13.8%)	51 (20.2%)
	Unknown	1 (0.2%)	0 (0)	1 (0.4%)
M	M0	380 (75.0%)	197 (77.6%)	183 (72.3%)
	M1	72 (14.2%)	31 (12.2%)	41 (16.2%)
	Unknown	55 (10.8%)	26 (10.2%)	29 (11.5%)

## Data Availability

Data is available at TCGA database (https://portal.gdc.cancer.gov/).

## Abbreviations

AUC: Area under the curve
BP: Biological process
CC: Cellular component
CRC: Colorectal cancer
CTLA-4: Cytotoxic T lymphocyte antigen-4
DCA: Decision curve analysis
GEO: Gene Expression Omnibus
GO: Gene Ontology
GSEA: Gene Set Enrichment Analysis
GDSC: Genomics of Drug Sensitivity in Cancer database
IPS: Immunophenoscore
KEGG: Kyoto Encyclopedia of Genes and Genomes
LASSO: Least absolute shrinkage and selection operator
LncRNA: Long non-coding RNA
MSI: Microsatellite instability
MSS: Microsatellite stability
MLKL: Mixed lineage kinase domain-like protein
MF: Molecular function
NRG: Necroptosis-related gene
nrlncRNA: Necroptosis-related lncRNA
OS: Overall survival
PCA: Principal component analysis
PD-L1: Programmed death ligand -1
PD-1: Programmed death receptor-1
ROC: Receiver operator characteristic
RIPK1: Receptor-interacting protein 1
RIPK3: Receptor-interacting protein 3
ssGSEA: Single sample Gene Set Enrichment Analysis
TCGA: The Cancer Genome Atlas
TCIA: The Cancer Immune Altas
IC50: The half-maximal inhibitory concentration
TME: Tumor microenvironment
TMB: Tumor mutation burden.

## Supplementary Materials

Supplementary 1 The survival curve of patients with high-expression and low-expression of 9 identified necroptosis-related lncRNAs.

Supplementary 2 Potential drugs prediction for high-risk group versus low-risk group.

## References

[1] Dekker E., Tanis P. J., Vleugels J., Kasi P. M., Wallace M. B. (2019). Colorectal cancer. *Lancet*.

[2] Siegel R. L., Miller K. D., Goding Sauer A. (2020). Colorectal cancer statistics, 2020. *CA: a Cancer Journal for Clinicians*.

[3] Benitez-Majano S., Fowler H., Maringe C., di Girolamo C., Rachet B. (2016). Deriving stage at diagnosis from multiple population-based sources: colorectal and lung cancer in England. *British Journal of Cancer*.

[4] Ganesh K., Stadler Z. K., Cercek A. (2019). Immunotherapy in colorectal cancer: rationale, challenges and potential. *Nature Reviews. Gastroenterology & Hepatology*.

[5] Hanahan D., Weinberg R. A. (2011). Hallmarks of cancer: the next generation. *Cell*.

[6] Fulda S. (2015). Targeting apoptosis for anticancer therapy. *Seminars in Cancer Biology*.

[7] Zhang P., Kawakami H., Liu W. (2018). Targeting CDK1 and MEK/ERK overcomes apoptotic resistance in BRAF-mutant human colorectal cancer. *Molecular Cancer Research*.

[8] Galluzzi L., Kroemer G. (2008). Necroptosis: a specialized pathway of programmed necrosis. *Cell*.

[9] Declercq W., Vanden B. T., Vandenabeele P. (2009). RIP kinases at the crossroads of cell death and survival. *Cell*.

[10] He G. W., Günther C., Thonn V. (2017). Regression of apoptosis-resistant colorectal tumors by induction of necroptosis in mice. *The Journal of Experimental Medicine*.

[11] Su Z., Yang Z., Xie L., DeWitt J. P., Chen Y. (2016). Cancer therapy in the necroptosis era. *Cell Death and Differentiation*.

[12] Kaczmarek A., Vandenabeele P., Krysko D. V. (2013). Necroptosis: the release of damage-associated molecular patterns and its physiological relevance. *Immunity*.

[13] Strilic B., Yang L., Albarrán-Juárez J. (2016). Tumour-cell-induced endothelial cell necroptosis via death receptor 6 promotes metastasis. *Nature*.

[14] McCormick K. D., Ghosh A., Trivedi S. (2016). Innate immune signaling through differential RIPK1 expression promote tumor progression in head and neck squamous cell carcinoma. *Carcinogenesis*.

[15] Snyder A. G., Hubbard N. W., Messmer M. N. (2019). Intratumoral activation of the necroptotic pathway components RIPK1 and RIPK3 potentiates antitumor immunity. *Sci Immunol*.

[16] Yao R. W., Wang Y., Chen L. L. (2019). Cellular functions of long noncoding RNAs. *Nature Cell Biology*.

[17] Di Martino M. T., Riillo C., Scionti F. (2021). miRNAs and lncRNAs as novel therapeutic targets to improve cancer immunotherapy. *Cancers (Basel)*.

[18] Kaller M., Gotz U., Hermeking H. (2017). Loss of p53-inducible long non-coding RNA LINC01021 increases chemosensitivity. *Oncotarget*.

[19] Zhai H., Fesler A., Schee K., Fodstad Ø., Flatmark K., Ju J. (2013). Clinical significance of long intergenic noncoding RNA-p21 in colorectal cancer. *Clinical Colorectal Cancer*.

[20] Tran D. D. H., Kessler C., Niehus S. E., Mahnkopf M., Koch A., Tamura T. (2018). Myc target gene, long intergenic noncoding RNA, _Linc00176_ in hepatocellular carcinoma regulates cell cycle and cell survival by titrating tumor suppressor microRNAs. *Oncogene*.

[21] Wang Z., Jensen M. A., Zenklusen J. C. (2016). A practical guide to the Cancer Genome Atlas (TCGA). *Methods in Molecular Biology*.

[22] Subramanian A., Tamayo P., Mootha V. K. (2005). Gene set enrichment analysis: a knowledge-based approach for interpreting genome-wide expression profiles. *Proceedings of the National Academy of Sciences of the United States of America*.

[23] Mootha V. K., Lindgren C. M., Eriksson K. F. (2003). PGC-1*α*-responsive genes involved in oxidative phosphorylation are coordinately downregulated in human diabetes. *Nature Genetics*.

[24] Zhao Z., Liu H., Zhou X. (2021). Necroptosis-related lncRNAs: predicting prognosis and the distinction between the cold and hot tumors in gastric cancer. *Journal of Oncology*.

[25] Li T., Fu J., Zeng Z. (2020). TIMER2.0 for analysis of tumor-infiltrating immune cells. *Nucleic Acids Research*.

[26] Charoentong P., Finotello F., Angelova M. (2017). Pan-cancer immunogenomic analyses reveal genotype-immunophenotype relationships and predictors of response to checkpoint blockade. *Cell Reports*.

[27] Maeser D., Gruener R. F., Huang R. S. (2021). oncoPredict: an R package for predicting in vivo or cancer patient drug response and biomarkers from cell line screening data. *Briefings in Bioinformatics*.

[28] Koboldt D. C., Zhang Q., Larson D. E. (2012). VarScan 2: somatic mutation and copy number alteration discovery in cancer by exome sequencing. *Genome Research*.

[29] Yuan R., Hou Y., Sun W. (2017). Natural products to prevent drug resistance in cancer chemotherapy: a review. *Annals of the New York Academy of Sciences*.

[30] Tonnus W., Meyer C., Paliege A. (2019). The pathological features of regulated necrosis. *The Journal of Pathology*.

[31] Huang Y., Zou Y., Xiong Q. (2021). Development of a novel necroptosis-associated miRNA risk signature to evaluate the prognosis of colon cancer patients. *Ann Transl Med*.

[32] Han Y., Wu N., Jiang M. (2019). Long non-coding RNA MYOSLID functions as a competing endogenous RNA to regulate MCL-1 expression by sponging miR-29c-3p in gastric cancer. *Cell Proliferation*.

[33] Xiong H. G., Li H., Xiao Y. (2019). Long noncoding RNA MYOSLID promotes invasion and metastasis by modulating the partial epithelial-mesenchymal transition program in head and neck squamous cell carcinoma. *Journal of Experimental & Clinical Cancer Research*.

[34] Xiao J., Liu Y., Yi J., Liu X. (2021). LINC02257, an enhancer RNA of prognostic value in colon adenocarcinoma, correlates with multi-omics immunotherapy-related analysis in 33 cancers. *Frontiers in Molecular Biosciences*.

[35] Xue Z., Cui C., Liao Z. (2018). Identification of LncRNA Linc00513 containing lupus-associated genetic variants as a novel regulator of interferon signaling pathway. *Frontiers in Immunology*.

[36] Cox T. R. (2021). The matrix in cancer. *Nature Reviews. Cancer*.

[37] Tian C., Clauser K. R., Öhlund D. (2019). Proteomic analyses of ECM during pancreatic ductal adenocarcinoma progression reveal different contributions by tumor and stromal cells. *Proceedings of the National Academy of Sciences of the United States of America*.

[38] de Visser K. E., Eichten A., Coussens L. M. (2006). Paradoxical roles of the immune system during cancer development. *Nature Reviews. Cancer*.

[39] Grivennikov S. I., Greten F. R., Karin M. (2010). Immunity, inflammation, and cancer. *Cell*.

[40] Palucka A. K., Coussens L. M. (2016). The basis of oncoimmunology. *Cell*.

[41] Schreiber R. D., Old L. J., Smyth M. J. (2011). Cancer immunoediting: integrating immunity’s roles in cancer suppression and promotion. *Science*.

[42] Zhang Q. W., Liu L., Gong C. Y. (2012). Prognostic significance of tumor-associated macrophages in solid tumor: a meta-analysis of the literature. *PLoS One*.

[43] Sasidharan N. V., Elkord E. (2018). Immune checkpoint inhibitors in cancer therapy: a focus on T-regulatory cells. *Immunology and Cell Biology*.

[44] Sawant D. V., Yano H., Chikina M. (2019). Adaptive plasticity of IL-10^+^ and IL-35^+^ T_reg_ cells cooperatively promotes tumor T cell exhaustion. *Nature Immunology*.

[45] Akeus P., Szeponik L., Ahlmanner F. (2018). Regulatory T cells control endothelial chemokine production and migration of T cells into intestinal tumors of APCmin/+ mice. *Cancer Immunology, Immunotherapy*.

[46] Gulubova M. V., Ananiev J. R., Vlaykova T. I., Yovchev Y., Tsoneva V., Manolova I. M. (2012). Role of dendritic cells in progression and clinical outcome of colon cancer. *International Journal of Colorectal Disease*.

[47] Xie Z. J., Jia L. M., He Y. C., Gao J. T. (2006). Morphological observation of tumor infiltrating immunocytes in human rectal cancer. *World Journal of Gastroenterology*.

[48] Tosolini M., Kirilovsky A., Mlecnik B. (2011). Clinical impact of different classes of infiltrating T cytotoxic and helper cells (Th1, th2, treg, th17) in patients with colorectal cancer. *Cancer Research*.

[49] Souza-Fonseca-Guimaraes F., Cursons J., Huntington N. D. (2019). The emergence of natural killer cells as a major target in cancer immunotherapy. *Trends in Immunology*.

[50] de Miguel M., Calvo E. (2020). Clinical challenges of immune checkpoint inhibitors. *Cancer Cell*.

[51] Seifert L., Werba G., Tiwari S. (2016). The necrosome promotes pancreatic oncogenesis via CXCL1 and Mincle-induced immune suppression. *Nature*.

[52] Xu Y., Lin Z., Zhao N. (2014). Receptor interactive protein kinase 3 promotes Cisplatin-triggered necrosis in apoptosis-resistant esophageal squamous cell carcinoma cells. *PLoS One*.

[53] Jing L., Song F., Liu Z. (2018). MLKL-PITP*α* signaling-mediated necroptosis contributes to cisplatin-triggered cell death in lung cancer A549 cells. *Cancer Letters*.

[54] Dunai Z. A., Imre G., Barna G. (2012). Staurosporine induces necroptotic cell death under caspase-compromised conditions in U937 cells. *PLoS One*.

[55] Addeo A., Banna G. L., Weiss G. J. (2019). Tumor mutation burden-from doubts to concerns-in reply. *JAMA Oncology*.

[56] Innocenti F., Ou F. S., Qu X. (2019). Mutational analysis of patients with colorectal cancer in CALGB/SWOG 80405 identifies new roles of microsatellite instability and tumor mutational burden for patient outcome. *Journal of Clinical Oncology*.

[57] Liu C., Liu D., Wang F. (2022). The interferon gamma-related long noncoding RNA signature predicts prognosis and indicates immune microenvironment infiltration in colon adenocarcinoma. *Frontiers in Oncology*.

