# Supplementary figures and images for: The Prediction of Necroptosis-Related lncRNAs in Prognosis and Anticancer Therapy of Colorectal Cancer
Source: Anal Cell Pathol (Amst). 2022 Sep 23;2022:7158684. doi: 10.1155/2022/7158684 (PMC9527116; doi:10.1155/2022/7158684)

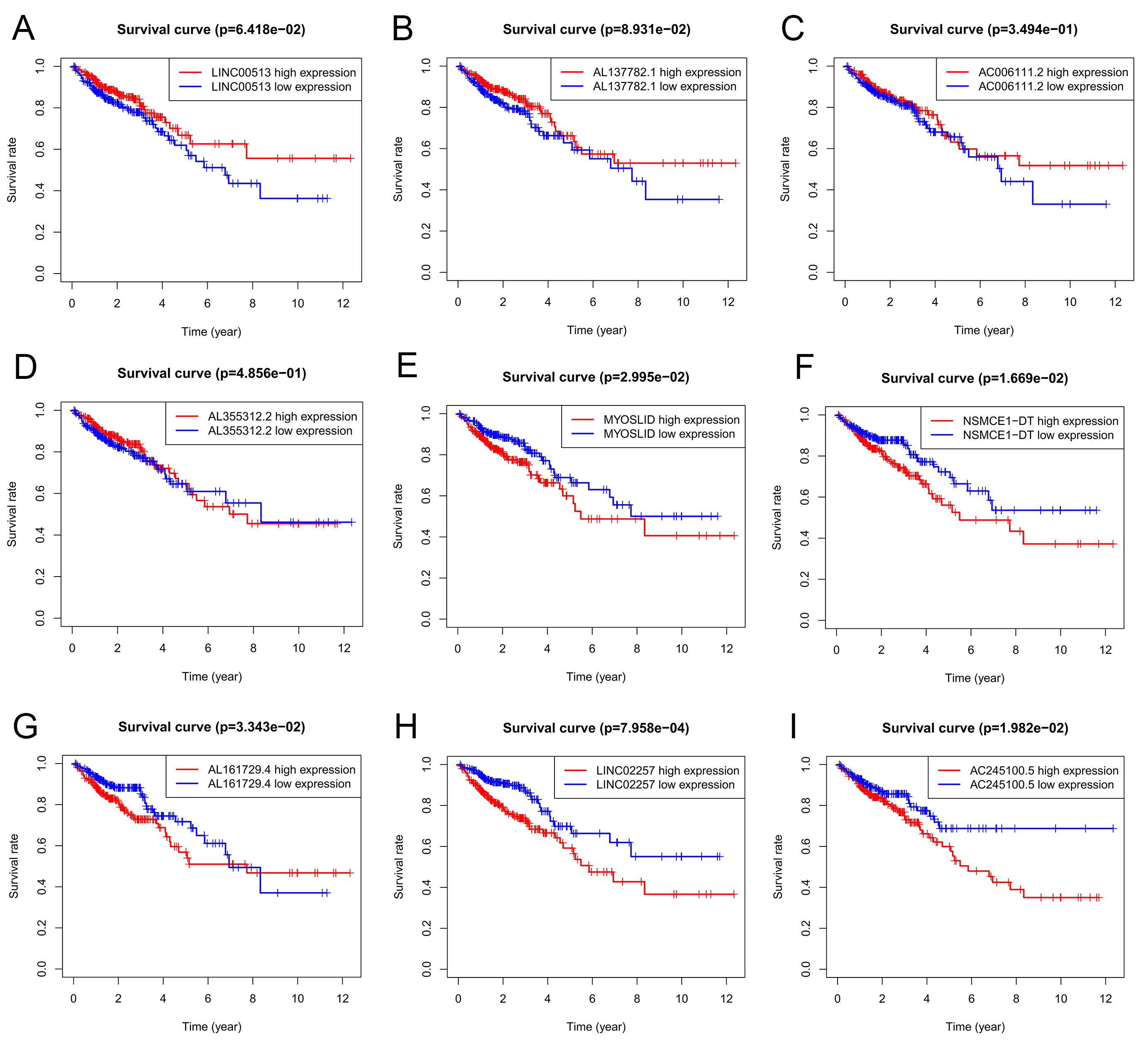

Supplement: Supplementary 1 — The survival curve of patients with high-expression and low-expression of 9 identified necroptosis-related lncRNAs. [file 7158684.f1.png]
