# Supplementary material for: The Prediction of Necroptosis-Related lncRNAs in Prognosis and Anticancer Therapy of Colorectal Cancer
Source: Anal Cell Pathol (Amst). 2022 Sep 23;2022:7158684. doi: 10.1155/2022/7158684 (PMC9527116; doi:10.1155/2022/7158684)
Supplement: Supplementary 2 — Potential drugs prediction for high-risk group versus low-risk group. [file 7158684.f2.pdf]

drug	avg(high-risk group)	avg(low-risk group)	p	fc	log2fc
Camptothecin_1003	NA	NA	0.23753	NA	NA
Vinblastine_1004	NA	0.083596891	0.47178	NA	NA
Cisplatin_1005	13742842.6	346359897.6	0.02008	25.2029	4.65552
Cytarabine_1006	111954.3671	36442.63069	0.71393	0.32551	-1.6192
Docetaxel_1007	NA	36.82528058	0.11013	NA	NA
Gefitinib_1010	46894.72374	NA	0.9226	NA	NA
Navitoclax_1011	864.7569497	221.4831301	0.84993	0.25612	-1.9651
Vorinostat_1012	21899557.57	30.77922586	0.20763	1.41E-06	-19.441
Nilotinib_1013	NA	NA	0.65287	NA	NA
Olaparib_1017	163473200.6	18569226.07	0.53135	0.11359	-3.1381
AZD7762_1022	38121607	865081.2063	0.88241	0.02269	-5.4616
Afatinib_1032	139.4259399	93.62781446	0.51083	0.67152	-0.5745
Staurosporine_1034	0.177569285	0.419797212	0.01021	2.36413	1.24131
PLX-4720_1036	NA	NA	0.8356	NA	NA
Wee1 Inhibitor_1046	NA	NA	0.00969	NA	NA
Nutlin-3a (-)_1047	763.0000752	437.9970392	0.00627	0.57405	-0.8008
Mirin_1048	NA	NA	0.31503	NA	NA
PD173074_1049	3816.063269	NA	0.51591	NA	NA
Alisertib_1051	236.5492888	12.03422161	0.02804	0.05087	-4.2969
MK-2206_1053	4655.550394	1080.60313	0.70173	0.23211	-2.1071
Palbociclib_1054	383.4511874	111.4871898	0.78296	0.29075	-1.7822
Dactolisib_1057	NA	NA	0.50033	NA	NA
Pictilisib_1058	496.5485619	8.290267079	0.78249	0.0167	-5.9044
PD0325901_1060	NA	NA	0.31821	NA	NA
Obatoclax Mesylate_1068	1.62E+19	12.35619919	0.55349	7.62E-19	-60.186
5-Fluorouracil_1073	1429467649	2127.981453	0.2116	1.49E-06	-19.358
Dasatinib_1079	5.366096987	6.327472783	0.00011	1.17916	0.23776
Paclitaxel_1080	NA	300.40032	0.24156	NA	NA
Crizotinib_1083	40.62656457	32.59129587	0.00702	0.80222	-0.3179
Rapamycin_1084	8107.152122	8096.855738	0.19583	0.99873	-0.0018
Sorafenib_1085	NA	NA	0.13223	NA	NA
Irinotecan_1088	NA	NA	0.38762	NA	NA
Oxaliplatin_1089	337818297	66770699.55	0.19688	0.19765	-2.339
BMS-536924_1091	8.649862931	8.597033153	0.31784	0.99389	-0.0088
GSK1904529A_1093	3836.983774	404.7273786	0.03718	0.10548	-3.245
PRIMA-1MET_1131	210367971.6	327198039.6	0.61821	1.55536	0.63725
Erlotinib_1168	2172.264293	1991.519418	0.91029	0.91679	-0.1253
Niraparib_1177	673.065369	152.1023224	0.085	0.22598	-2.1457
MK-1775_1179	90681757.82	5195.264908	0.49266	5.73E-05	-14.091
Dinaciclib_1180	NA	NA	0.38149	NA	NA
Gemcitabine_1190	NA	NA	0.70092	NA	NA
Bortezomib_1191	NA	NA	0.36106	NA	NA
Tamoxifen_1199	NA	NA	0.00124	NA	NA
Fulvestrant_1200	21.82218045	25.87006116	0.0001	1.18549	0.24549
EPZ004777_1237	1.94E+14	343.7214719	0.45114	1.77E-12	-39.042
YK-4-279_1239	NA	3972.705113	0.59562	NA	NA
BMS-345541_1249	NA	NA	0.34709	NA	NA
AZ960_1250	1948793.892	653421.9478	0.1433	0.3353	-1.5765
Talazoparib_1259	39003.20016	NA	0.57139	NA	NA
XAV939_1268	125.0078603	98.75604912	0.79558	0.79	-0.3401
Trametinib_1372	2.202849839	1.997576271	0.00401	0.90681	-0.1411
Dabrafenib_1373	NA	NA	0.1593	NA	NA
Temozolomide_1375	652.3975836	699.1420401	0.0054	1.07165	0.09983
AZD5438_1401	NA	3518.183238	0.07436	NA	NA
IAP_5620_1428	1006.458898	370.9794567	0.79887	0.3686	-1.4399
AZD2014_1441	NA	NA	0.32611	NA	NA
AZD1208_1449	1870067.841	1309597.479	0.40002	0.70029	-0.514

AZD1332_1463	5962.301821	551.1330853	0.55308	0.09244	-3.4354
Ruxolitinib_1507	196.7439194	255.0192129	0.00355	1.2962	0.37429
Linsitinib_1510	68.77880757	85.09258729	0.13694	1.23719	0.30707
Epirubicin_1511	NA	NA	0.39797	NA	NA
Cyclophosphamide_1512	23162.01773	227.8764583	0.0255	0.00984	-6.6674
Pevonedistat_1529	NA	NA	0.96473	NA	NA
Sapitinib_1549	2130.321265	1883.177638	0.34784	0.88399	-0.1779
Uprosertib_1553	2327.904315	46.20293343	0.05527	0.01985	-5.6549
LCL161_1557	284.9227229	246.4671915	0.46142	0.86503	-0.2092
Lapatinib_1558	1.19E+15	6.83E+13	0.19416	0.05763	-4.1171
Luminespib_1559	NA	NA	0.33574	NA	NA
Alpelisib_1560	147.5038978	70.88120269	0.05747	0.48054	-1.0573
Taselisib_1561	36100.93055	16.62382789	0.99879	0.00046	-11.085
EPZ5676_1563	2.76E+12	20599784438	0.87523	0.00747	-7.0656
SCH772984_1564	NA	NA	0.6241	NA	NA
IWP-2_1576	46.14036583	30.17844361	0.20049	0.65406	-0.6125
Leflunomide_1578	221.4272917	231.9705149	0.19416	1.04761	0.06711
Entinostat_1593	NA	28.67083328	0.0176	NA	NA
LGK974_1598	90.38689437	117.4237646	0.00061	1.29912	0.37754
VE-822_1613	4770752.099	1142067.102	0.34598	0.23939	-2.0626
WZ4003_1614	1724.088178	974.621105	0.01433	0.5653	-0.8229
CZC24832_1615	240.6817035	251.8537309	0.47183	1.04642	0.06546
AZD5582_1617	962215.7678	823231.9202	0.13376	0.85556	-0.2251
GSK2606414_1618	54.4701258	56.66651912	0.50267	1.04032	0.05703
PFI3_1620	23370.28644	1508.561478	0.27624	0.06455	-3.9534
PCI-34051_1621	518.5090238	232.4778158	0.0249	0.44836	-1.1573
Wnt-C59_1622	219.8031051	93.44422844	0.01636	0.42513	-1.234
I-BET-762_1624	43.04231883	55.81344223	0.05527	1.29671	0.37486
RVX-208_1625	148.3506185	156.5610961	0.01487	1.05535	0.07771
OTX015_1626	44.08884015	65.12790744	0.45553	1.4772	0.56286
GSK343_1627	1514.130393	338.5292576	0.2819	0.22358	-2.1611
ML323_1629	150.0850124	129.9711328	0.9262	0.86598	-0.2076
Entospletinib_1630	154.4489737	62.44755306	0.06242	0.40432	-1.3064
PRT062607_1631	45.22855751	51.08031019	0.00287	1.12938	0.17553
AGI-6780_1634	135.8663244	280.1738543	0.01925	2.06213	1.04413
Picolinici-acid_1635	319.7628108	300.3143074	0.41307	0.93918	-0.0905
AZD5153_1706	18.05279154	20.2304796	0.94358	1.12063	0.16431
CDK9_5576_1708	NA	NA	0.54956	NA	NA
CDK9_5038_1709	NA	NA	0.73545	NA	NA
Eg5_9814_1712	NA	NA	0.45896	NA	NA
ERK_2440_1713	NA	NA	0.5336	NA	NA
ERK_6604_1714	106.6749181	NA	0.41208	NA	NA
IRAK4_4710_1716	1127.004388	485.627409	0.9891	0.4309	-1.2146
JAK1_8709_1718	22700.78525	759.7274378	0.00812	0.03347	-4.9011
AZD5991_1720	NA	NA	0.65453	NA	NA
PAK_5339_1730	17.43053222	13.85656476	0.00131	0.79496	-0.331
TAF1_5496_1732	125.5536384	95.03764152	0.72119	0.75695	-0.4017
ULK1_4989_1733	992217.5353	55.57313744	0.90836	5.60E-05	-14.124
VSP34_8731_1734	NA	NA	0.07517	NA	NA
Selumetinib_1736	NA	NA	0.40859	NA	NA
IGF1R_3801_1738	451892541.3	1430232512	0.21049	3.16498	1.6622
JAK_8517_1739	1200231439	154.6185651	0.18777	1.29E-07	-22.888
AZD4547_1786	697867.9674	192762.8948	0.65154	0.27622	-1.8561
Ibrutinib_1799	40105.15306	22995.2979	0.1952	0.57338	-0.8024
Zoledronate_1802	537.6120563	64.17700047	0.11958	0.11937	-3.0664
Acetalax_1804	4667.030893	2731.89713	0.0998	0.58536	-0.7726
Oxaliplatin_1806	6983.655159	2249.494979	0.78436	0.32211	-1.6344
Carmustine_1807	1417.725718	942.1797339	0.00075	0.66457	-0.5895

Topotecan_1808	NA	NA	0.09047	NA	NA
Teniposide_1809	1.88E+20	8.03E+15	0.2264	4.27E-05	-14.516
Mitoxantrone_1810	3.77E+19	6.91E+16	0.46438	0.00183	-9.0936
Dactinomycin_1811	358697114.5	1.201194016	0.32168	3.35E-09	-28.154
Fludarabine_1813	NA	NA	0.0005	NA	NA
Nelarabine_1814	614.8708801	689.9068295	0.00476	1.12204	0.16612
Fulvestrant_1816	1275.883209	272.6767378	0.40651	0.21372	-2.2262
Vincristine_1818	NA	1.58E+16	0.01214	NA	NA
Docetaxel_1819	NA	NA	0.02169	NA	NA
phyllotoxin bromide_1825	NA	3830.638914	0.15874	NA	NA
Dihydrorotenone_1827	3.675732228	2.97749173	0.00462	0.81004	-0.3039
Gallibiscoquinazole_1830	1.02E+11	31.70975573	0.05933	3.10E-10	-31.587
Elephantin_1835	NA	NA	0.77679	NA	NA
Sinularin_1838	2357790.318	1661110.483	0.53494	0.70452	-0.5053
Sabutoclax_1849	NA	NA	0.43757	NA	NA
LY2109761_1852	259.3201058	287.742568	0.00276	1.1096	0.15004
OF-1_1853	96.05613972	130.0562261	0.08567	1.35396	0.43719
MN-64_1854	112.4499647	125.5694085	0.01251	1.11667	0.1592
(G12C) Inhibitor-12_1855	47214732.63	173.0202155	0.16376	3.66E-06	-18.058
MG-132_1862	NA	NA	0.34156	NA	NA
BDP-00009066_1866	NA	NA	0.04837	NA	NA
Buparlisib_1873	NA	NA	0.9794	NA	NA
Ulixertinib_1908	1948.807527	242.2233386	0.00119	0.12429	-3.0082
Venetoclax_1909	NA	NA	0.00131	NA	NA
ABT737_1910	NA	16.58286366	0.44301	NA	NA
Dactinomycin_1911	NA	NA	0.69029	NA	NA
Afuresertib_1912	21.44550294	28.609079	0.09586	1.33404	0.4158
AGI-5198_1913	211.1194825	172.3902413	0.11573	0.81655	-0.2924
AZD3759_1915	103.9158501	82.28274603	0.87954	0.79182	-0.3368
AZD5363_1916	2406.063824	44.12501229	0.44641	0.01834	-5.7689
AZD6738_1917	NA	NA	0.158	NA	NA
AZD8186_1918	28297.59279	88.64511885	0.00416	0.00313	-8.3184
Osimertinib_1919	15523.45534	1568.965363	0.53854	0.10107	-3.3066
Cediranib_1922	NA	3.37E+11	0.27408	NA	NA
Ipatasertib_1924	411.4741209	48.41228319	0.58982	0.11766	-3.0874
GDC0810_1925	617.0891446	534.5072074	0.82998	0.86618	-0.2073
GNE-317_1926	10.29091389	3.686383497	0.30592	0.35822	-1.4811
GSK2578215A_1927	7071.230356	865.419253	0.62981	0.12239	-3.0305
I-BRD9_1928	119791.5366	7954.534326	0.62035	0.0664	-3.9126
omerase Inhibitor IX_1930	NA	10.02674138	0.12482	NA	NA
MIRA-1_1931	644.7629728	645.2201617	0.07175	1.00071	0.00102
NVP-ADW742_1932	56384.4589	11552.37428	0.87331	0.20489	-2.2871
P22077_1933	2.96E+12	2.78E+11	0.17334	0.09387	-3.4132
Savolitinib_1936	75.65466695	32.43681669	0.00741	0.42875	-1.2218
UMI-77_1939	NA	NA	0.24505	NA	NA
WIKI4_1940	252275958.5	1684.463427	0.47483	6.68E-06	-17.192
antronium bromide_1941	0.057291254	0.020849789	8.90E-06	0.36393	-1.4583
MIM1_1996	126.2428762	140.7849629	0.43382	1.11519	0.15729
WEHI-539_1997	NA	69.98919494	0.26693	NA	NA
BPD-00008900_1998	121.2197015	157.9722396	0.0539	1.30319	0.38205
Foretinib_2040	3.144993446	3.229707181	0.83804	1.02694	0.03835
BIBR-1532_2043	201.083485	309.9801702	0.00688	1.54155	0.62438
Pyridostatin_2044	NA	NA	0.9758	NA	NA
AMG-319_2045	323507.9331	275.0780946	5.25E-07	0.00085	-10.2
MK-8776_2046	30686.42554	31526.45347	0.29909	1.02737	0.03896
Ulixertinib_2047	19.98201506	19.02593843	0.53734	0.95215	-0.0707
Vinorelbine_2048	NA	8.850537943	0.23167	NA	NA
VX-11e_2096	160.7662387	84.83791763	0.12472	0.52771	-0.9222

Uprosertib_2106	33540.16273	34.3505449	0.04945	0.00102	-9.9313
LJI308_2107	1485.821935	392.581894	0.76899	0.26422	-1.9202
AZ6102_2109	NA	NA	0.6966	NA	NA
GSK591_2110	127.1828689	176.9451129	0.00132	1.39127	0.4764
VE821_2111	4549.420972	4660.094471	0.32734	1.02433	0.03468
AT13148_2170	285.8398128	472.9119759	0.0545	1.65447	0.72636
